# A review of the vector management methods to prevent and control outbreaks of West Nile virus infection and the challenge for Europe

**DOI:** 10.1186/1756-3305-7-323

**Published:** 2014-07-11

**Authors:** Romeo Bellini, Herve Zeller, Wim Van Bortel

**Affiliations:** 1 Centro Agricoltura Ambiente “G.Nicoli”, Via Argini Nord 3351, Crevalcore 40014, Italy; 2 European Centre for Disease Prevention and Control, Stockholm, Sweden

**Keywords:** West Nile virus, Vector-borne disease, Vector surveillance, Vector control, Europe

## Abstract

West Nile virus infection is a growing concern in Europe. Vector management is often the primary option to prevent and control outbreaks of the disease. Its implementation is, however, complex and needs to be supported by integrated multidisciplinary surveillance systems and to be organized within the framework of predefined response plans. The impact of the vector control measures depends on multiple factors and the identification of the best combination of vector control methods is therefore not always straightforward. Therefore, this contribution aims at critically reviewing the existing vector control methods to prevent and control outbreaks of West Nile virus infection and to present the challenges for Europe.

Most West Nile virus vector control experiences have been recently developed in the US, where ecological conditions are different from the EU and vector control is organized under a different regulatory frame. The extrapolation of information produced in North America to Europe might be limited because of the seemingly different epidemiology in the European region. Therefore, there is an urgent need to analyse the European experiences of the prevention and control of outbreaks of West Nile virus infection and to perform robust cost-benefit analysis that can guide the implementation of the appropriate control measures. Furthermore, to be effective, vector control programs require a strong organisational backbone relying on a previously defined plan, skilled technicians and operators, appropriate equipment, and sufficient financial resources. A decision making guide scheme is proposed which may assist in the process of implementation of vector control measures tailored on specific areas and considering the available information and possible scenarios.

## Introduction

West Nile virus (WNV) infection is a viral disease transmitted by mosquitoes and distributed worldwide. The virus is primarily maintained in an enzootic cycle involving a number of wild birds and ornithophilic mosquito species. When the climatic or environmental conditions become favourable the virus circulation may increase and transmission to other susceptible hosts such as humans and equids may occur. These are considered dead-end hosts because the viremia remains at a level not suitable for mosquito infection [1,2]. The vast majority of human cases remain asymptomatic and severe neuroinvasive illness is reported in less than 1% of infected people [3,4]. The high number of non-symptomatic cases may increase the risk of WNV transmission through blood donation or organ transplants. WNV can be transported over long distances in migratory birds. While resting in wetland areas they might come in contact with the local mosquito species, and possibly initiate a local amplification cycle. In temperate regions the virus may overwinter in female mosquitoes as well as in birds, so there is no need for continuous re-introductions [5-10].

WNV infection is a growing concern in Europe. The infection has been documented since the early 1950s in several countries of the European Union (EU) [11]. In 1996, Romania reported the largest outbreak of WNV infection in humans to date inside the EU, with over 390 confirmed cases [12]. From 2000, human cases and outbreaks in the EU and Balkan countries have been reported in Albania, Austria, Bosnia and Herzegovina, Bulgaria, Croatia, Czech Republic, The Former Yugoslav Republic of Macedonia, France, Greece, Hungary, Italy, Kosovo, Montenegro, Portugal, Romania, Serbia, and Spain [13-27]. In Europe WNV has been found in several field collected mosquito species, such as *Culex pipiens s.l*., *Culex modestus*, *Culex univittatus*, *Coquillettidia richiardii*, *Aedes cantans*, *Aedes caspius*, *Aedes excrucians*, *Aedes vexans*, *Anopheles maculipennis* s.s. and *Anopheles atroparvus*[11,28-32], but based on the current evidence the major vector role in outbreaks of WNV infection in Europe seems to be covered by *Cx. pipiens* s.l. [12,31,33] with *Cx. modestus* playing a role in specific regions [34].

The prevention and control of outbreaks of WNV infection are complex and need integrated multidisciplinary surveillance systems and response plans [35]. The integrated vector management (IVM) is the primary option to prevent and control outbreaks. The WHO defines IVM as “a rational decision-making process for the optimal use of resources for vector control” [36]. IVM is intended to utilize the best cost-benefit combination of all available control methods in a sustainable way respecting the environment in order to reduce the vector density and/or the vector-human contacts to levels not posing a public health concern. Yet, the implementation of IVM is hampered by the complexity of the WNV transmission cycle, the difficulties in estimating the size of the forthcoming outbreak in a timely matter and the related risk assessment. Moreover, the information necessary to make a robust cost-benefit analysis that guides the implementation of the appropriate measures is largely missing, as well as the species-specific WNV vector density thresholds, and the fact that mosquito control programs mostly rely on empirical data and past experience. The identification of the best vector control methods is therefore not always straightforward. Hence, this contribution aims at reviewing the existing vector control methods to prevent and control outbreaks of WNV infection and to present the challenges in a European context.

## Review

The data bases PubMed and Promed were searched using the key words: West Nile, West Nile control, West Nile prevention, West Nile and cost benefit, *Culex pipiens*, *Culex* and overwintering, *Culex pipiens* and resistance, adulticide, aerial spray, larval control, larval management, mass trap, overwintering, ULV. The most important relevant accessible reports and unpublished documents were consulted and expert colleagues were contacted to provide additional information.

In the context of the EU WNV and blood safety preparedness plan an affected area is defined as an area with at least one case of WNV infection in humans according to the EU case definition [37]. Assigning an area as an affected area triggers the implementation of control measures. Hence this trigger, one case of WNV infection according to the EU case definition, was used as a definition of an outbreak in this paper. Prevention of WNV infection is intended before the occurrence of equine or human cases, while control of WNV infection is considered following the occurrence of at least one case of WNV infection.

### Control measures targeting the mosquito larvae

In the context of outbreak prevention of WNV infection the objective of larval control is to contain immature stages in order to keep adult mosquito populations at density levels below which they pose a public health risk. Unfortunately, insufficient information exists to establish a larval density threshold that can be used to guide control operations [38-40]. The role of larval control to contain outbreaks of WNV infection should be considered as part of an IVM program (see Vector control decision making process section). Hence a larval control program must be in place in the areas known as susceptible to outbreaks of WNV infection (see Vector control decision making process section, Table 1).

**Table 1:** Overview of the vector control methods currently used for the prevention and control of outbreaks of WNV infection

			**Suggested use**^**3**^	
**Activities**	**Target environments**	**Products and equipment**	**Prevention**	**Outbreak control**
**Targeting the mosquito larvae**^**1**^				
Environmental management and source reduction	artificial wetlands, hunting farms, lagoons, recreational, rice fields, irrigation canals, urban environments	Ground equipment, excavators, mowers, pumps	+++	Part of IVM programme
Chemical	Breeding sites as specified in the insecticide label	According to WHO guidelines and EU regulations:	++	Part of IVM programme
		S-Methoprene, Diflubenzuron, Pyriproxyfen, Triflumuron		
Biological	artificial wetlands, lagoons, rice fields, irrigation canals, urban environments	Gambusia (where allowed), native fish species, *Macrocyclops*	++	Part of IVM programme
Microbial and others	wetlands, lagoons, rice fields, irrigation canals, urban environments	According to WHO guidelines and EU regulations	++	Part of IVM programme
		*Bacillus thuringiensis israelensis*, *Bacillus sphaericus*, Spinosad		
Community participation	inhabited areas	Insecticide formulations for domestic use	+	Part of IVM programme
**Targeting the adult mosquitoes**^**2**^				
Aerial adulticide treatments	Outbreak area	Aerial ULV	Not allowed	Not allowed (specific exemption in extraordinary cases only)
Ground adulticide treatments (ULV or LV)	Outdoors, outbreak area	According to WHO guidelines and EU regulations	Not suggested	++
Mass trapping	domestic use in inhabited areas	attractive traps selective for mosquito	-	-
Personal protection measures	any environment during mosquito activity peak in case of West Nile virus risk	mosquito screen over windows, space or topical repellents, protective clothes	+++	+++

^1^The suggested use of vector control methods targeting the mosquito larvae refers to the impact on the vector population. The impact in reducing the WNV transmission is not well known; ^2^The suggested use of vector control methods targeting the adult mosquitoes refers to the impact on WNV transmission; ^3^+++ highly useful; ++ useful; + potentially useful in some circumstances; - currently no evidence of usefulness.

IVM: integrated Vector management; ULV: Ultra Low Volume.

#### Environmental management and source reduction

These activities have the important advantage over direct vector control operations to be long lasting or even permanent in their effects, thus allowing a positive cost-benefit balance. To appropriately adopt environmental management practices field investigations should be performed in advance to characterize and geo-reference breeding sites. Skilled technicians should conduct field inspections to cover at least one whole favourable season, mapping the potential and active breeding sites, defining their dimensions, accessibility, mosquito productivity by species, environmental value taking into account biodiversity issues especially when dealing with natural protected areas, seasonal dynamic, and indicating possible management actions. Source reduction can include simple activities such as the proper disposal of containers in backyards and the cleaning of rain gutters by property owners, to agronomic practices aimed at the reduction of standing waters, to extensive regional water management projects on public land. All these activities eliminate or substantially reduce mosquito breeding habitats and diminish the need for repeated application of insecticides [41-52].

#### Biological larval control

Direct biological larval control includes methods to enhance the activity of natural antagonists by introducing bio-control agents such as fishes and copepods or by allowing natural predators to colonise isolated water bodies by digging ditches to connect them [53-59]. This approach must be carefully evaluated in terms of environmental risk and the possible negative impact on biodiversity. Fish species such as *Gambusia spp*. are broad range predators and exotic to Europe. The “Habitats and Birds Directives” contain restrictions on the deliberate introductions of alien species into the wild and rearing and introduction of these species in water bodies is currently restricted in most countries. Predacious indigenous copepods such as *Macrocyclops albidus* may easily be reared and introduced in artificial containers where they reproduce and strongly reduce mosquito larval density [60]. They have limitations because they require good water quality and have been shown to be highly effective on *Aedes*, while only relatively effective on *Culex* species [58,61].

#### Naturally derived products and microbial larval control

Larval control by industrially produced bacteria is well developed because of the high efficacy combined with the selectivity of action which makes these products the best possible choice in terms of environmental impact. Several formulations based on *Bacillus thuringiensis israelensis* (*B.t.i*.) and *Bacillus sphaericus* (*B.s*.) bacteria formulations are available on the EU market or in the process of being marketed in the near future (see Biocides used in Europe section). The use of these products is usually allowed even in naturally protected areas. The main disadvantage is the short lasting activity which requires repeated applications. When bio-larvicide applications target large breeding areas (such as rice fields or wetlands) they may be performed by air (helicopter or fixed wings) following specific authorizations in exception of the 2002/2277 EU Parliament resolution. Relevant resistance phenomena have been evidenced following intensive utilization of *B.s*. against *Culex* species [62-64], while never observed on *B.t.i*. A new insecticide, Spinosad, derived via fermentation from the naturally occurring soil actinomycete, *Saccharopolyspora spinosa*, is also available in some countries and in the marketing process in others.

#### Chemical larval control

Direct larval control using chemical larvicides may be implemented using different products depending on the natural values of the target environment. Chemical larval control can only be done by ground application targeting previously mapped breeding sites. Four main active ingredients, all pertaining to the Insect Growth Regulators (IGRs) category, are currently available or under the revision process in the EU market: S-Methoprene, Diflubenzuron, Pyriproxyfen, Triflumuron [65]. Aerial application is not allowed following the adoption of the European Parliament resolution: “Towards a thematic strategy on the sustainable use of pesticides” (2002/2277(INI)) (see Biocides used in Europe section).

#### Community participation

*Culex* control by community participation includes the elimination of breeding sites and larval control in private areas and can be part of the environmental management and source reduction approaches. Regular information campaigns may be organized targeting the most relevant citizen groups (e.g. farmers, land owners, scholars) aimed to promote the adoption of appropriate behaviour. These basic information campaigns should clearly consider the messages and the timing to obtain the best possible efficacy both in situation of possible and already ongoing outbreaks without losing effectiveness.

### Control measures targeting the adult mosquito

In general adult mosquito control operations are aimed at the prompt reduction of adult mosquito density and longevity in a defined area in case of an outbreak. Adult control is not recommended as a routine method in outbreak prevention of WNV infection because outbreaks are currently largely unpredictable in time, space, and size. The environmental toxicity and broad action spectrum of the available insecticides for mosquito adult control implies that they can only be used in case of real need and focused on well-defined target areas. A well designed and managed active surveillance programme can be instrumental to define when adulticiding is appropriate (see Vector control decision making process section, Table 1). In any case measures of vector control to stop ongoing outbreaks of WNV infection should be implemented according to a pre-developed plan established as a public health measure by the sanitary authority. The plan should be technically detailed and responsibilities clearly defined.

#### Adult control

Adult control is considered the most convenient vector control approach during the outbreak phase because it has the capacity to promptly reduce infective mosquito females responsible for vectoring the disease as well as to reduce the mean longevity and the total reproduction capacity of local vector populations. The biocides and their application are strictly regulated by the EU and WHO provides guidelines on their use for public health purposes (see Biocides used in Europe section).

The current ground application technology for mosquito control mainly refers to equipment able to produce thermal or cold aerosols with particle dimensions of a defined range. It is considered that for mosquito adult control the best efficacy in terms of mortality is achieved when droplet diameter is in the range 5-30 μm (the best droplet size must be defined more precisely according to parameters such as formulation type and concentration, application equipment, environmental condition, habitat type, target species sensitivity) [66-71]. Ultra Low Volume (ULV) application is defined as the minimum effective volume of the formulated product without any further dilution (0.6-18 l/h). While Low Volume (LV) (18-60 l/h) and High Volume (HV) (>150 l/h) require the formulation being diluted before use.

There is no vector density threshold to guide decisions about the degree of vector population suppression that must be attained, or for how long this suppression must be maintained to reduce human disease transmission [38]. Through mathematical modelling it has been suggested that it would be appropriate to focus the vector control activities late in the season to target overwintering mosquitoes when temperatures are dropping [72]. The practical implications of a vector control program targeting overwintering mosquito females need further evaluation.

#### Mass trapping

A number of traps have been developed to attract, catch and kill large numbers of mosquitoes, thus removing them from the target area. This technology is developing rapidly and there is considerable variability in the way these traps function [73,74]. Studies set-up to determine the real level of protection provided by these traps in the area they possibly cover showed low effectiveness [75,76].

### Personal protection methods

Personal protection methods from mosquito bites include the use of space or topical repellents, the installation of mosquito screens over windows, wearing protective clothes (such as long sleeves and long pants), the adoption of preventative behaviour useful to help to avoid or reduce exposure to mosquito bites (i.e. limit outdoor activities when mosquitoes are most active), and mosquito proof homes. The effectiveness of personal protection methods in reducing the risk of acquiring WNV infection has been demonstrated by Loeb *et al*. [77] in Ontario, Canada. Between the different available tools for self-protection, repellents play an important role as they protect people when outdoors for normal activities. The most effective repellent substances are: DEET (N,N Diethyl Toluamide), Picaridin (1-(1methylpropylcarbonyl)-2-(hydroxyethyl)-piperidine), also known as KBR, and IR3535. Recently, various essential oils have been proposed as insect repellents, but their protection against anthropophilic species is considered low [78-82]. A number of studies have demonstrated that electronic mosquito repellers are ineffective and that some of them could even increase the biting frequency of mosquitoes [83,84].

To favour a large adoption of personal protection methods specific information campaigns must be conducted. These campaigns are not suggested as a routine preventative method for outbreaks of WNV infection, but they need to be connected to an active surveillance system in order to be activated only in case of real risk (see Vector control decision making process section).

### Biocides used in Europe

The biocides that can be used for larval and adult mosquito control are based on the active ingredients covered in the review being carried out under the Biocides Directive 98/8/EC (second phase of the 10-year work programme referred to in Article 16), Commission Regulation (EC) No 1451/2007 of 4 December 2007 and more recently by the Biocides Regulation 528/2012. The objective of the new Regulation is to improve the functioning of the EU internal market in biocidal products whilst ensuring a high level of environmental and human health protection. Some concerns have been raised on the possibility that following the procedure few products will available for vector control thus weakening our capacity to protect communities from vector-borne diseases.

WHO through the WHO Pesticide Evaluation Scheme (WHOPES) collects, consolidates, evaluates and disseminates information and guidelines on the use of pesticides for public health. Updated information can be found on the website (http://www.who.int/whopes/en/).

The organization of mosquito control refers to ground application as the aerial distribution of toxic insecticides is strictly forbidden following the European Parliament resolution 2002/2277. In case of an ongoing large epidemic there might be an emergency exception under the responsibility of regional or country authorities. As an example, in Greece, experimental aerial treatments using a helicopter equipped with ULV nozzles to spray pyrethroids (Deltamethrin and D-Phenothrin) against mosquitoes have been recently conducted during the outbreak of WNV infection [85].

For mosquito larval control five active ingredients are currently available or under the revision process, namely S-Methoprene, Diflubenzuron, Pyriproxyfen, Triflumuron, Spinosad [65]. Several formulations of microbial larvicides based on *Bacillus thuringiensis israelensis* are available on the EU market (liquid, powder, granular, briquette) [86]. *Bacillus sphaericus* formulations are present in some countries (i.e. France, Germany), while in the process of being marketed in the near future in other countries.

The main active ingredients for adult control belong to the pyrethrin and pyrethroids group. These compounds have good safety margins in terms of acute toxicity for warm blooded animals but are toxic for non target insect species and aquatic organisms [87-90]. Possible long term effects and cumulative effects are much more complex to establish.

### Impact and cost-benefit of vector management on WNV transmission

Available data on the impact of vector control on WNV transmission is available from the USA showing both failures and successes (summarised in Table 2), while documented examples for Europe are not available. In general, single application for adult control has been shown to have a transient effect and, in the event of an epidemic, multiple sequential treatments may be required to decrease vector abundance to a level that will stop transmission [Table 2 and [91,92]]. As a general opinion shared by US colleagues repeated aerial treatments seem to be more effective in reducing WNV circulation than ground application because the capacity to rapidly cover large areas. Effective surveillance must be maintained during vector control activities to determine if and when re-treatment is required to maintain suppression of the vector populations. Vegetation may have a negative effect on ULV treatment efficacy by filtering the aerosol leading to a reduction in the amount of pesticide available to impact the mosquito, and by reducing wind speed in the canopy thus reducing aerosol dispersion.

**Table 2:** Examples from the USA on the use of adult mosquito management in the control of outbreaks of WNV infection

**Study**	**Observed efficacy**	**Reference**
Louisiana	This study calculated an 86% decrease (compared with a 5-year average) in WNV mosquito vector species in 2002 resulting from increasing control efforts (aerial and ground ULV with Naled (1,2-dibromo-2,2-dichloroethyl dimethyl phosphate) over a 4-month period in St. Tammany Parish, Louisiana.	[93]
Florida	This study estimated a seasonal mean 64.1% *Culex nigripalpus* density reduction following emergency aerial sprays with Naled in 26 Florida counties during 2004, in response to several hurricanes.	[94]
Boston	Poor efficacy of ground ULV treatments with Resmethrin against *Cx. pipiens* and *Cx. restuans* in the Boston area showed that the aerosol plume delivered from the road failed to contact the target mosquitoes because it was blocked by the vegetation. Therefore, the application was unable to reduce transmission of WNV.	[95]
Kentucky	The University of Kentucky evaluated the efficacy of professional application of bifenthrin and lambda-cyhalothrin as barrier treatments with backpack mist blower directed to all vegetative surfaces up to the height of 3 m. Residual efficacy in reducing adult mosquito populations was studied at 24 residential properties (eight replications by three treatments). Mosquito populations were measured on each property by using five methods: CO_2_-baited CDC light traps (without a light), human landing rates, CDC gravid traps, ovitraps, and sweep nets. Populations were monitored weekly for two weeks before treatment and eight weeks post-treatment. Additionally, to confirm residual efficacy of each insecticide, a randomly treated leaf underwent a no-choice bioassay with laboratory-reared *Ae. albopictus*. Trap collections indicate *Ae. albopictus* and *Cx. pipiens* as the most abundant species in the area. Both insecticides significantly reduced *Ae. albopictus* density in comparison with the untreated control areas (85.1-89.5% reduction), and *Ae. albopictus* bioassay showed significant residual efficacy for both insecticides up to six weeks post-treatment. In contrast, *Culex* spp. were not reduced by either insecticides. The study seems to therefore indicate that barrier sprays applied to low-lying vegetation do not properly target adult daytime resting sites for *Culex* mosquitoes but that they can reduce *Aedes* mosquitoes. Perhaps *Culex* spp. abundance may be reduced by treating upper tree canopies.	[96]
California	In California, in 2004-2005, a program was implemented to control the amplification and dispersal of WNV using sequential ground ULV applications of Pyrenone® 25-5 (Insecticide containing Pyrethrins (5.0%) and Piperonyl Butoxide (25.0%)). Local treatments were started one month after the initial detection. Evaluations indicated that while the treatments were effective in reducing vector abundance, they had little effect on virus transmission, and WNV was dispersing throughout the area.	[97]
Sacramento	Carney *et al*. determined the efficacy of the aerial treatments with pyrethrins combined with piperonyl butoxide (PBO) on WNV transmission in Sacramento county during the 2005 epidemic. No human cases occurred in the treated area after repeated treatments (number of treatments not specified), compared with 18 cases in the untreated area. Consequently, they considered the emergency aerial spray to be effective in reducing both mosquito populations and WNV cases.	[98]
California	In the Coachella Valley good results were achieved with early season treatment in mid-April immediately following the first detection of WNV.	[99]
California	In Sacramento County, an aerial distribution of Evergreen EC 60-6 (insecticide containing Pyrethrins (6.00%) and Piperonyl butoxide (60.00%)) over approximately 215 km^2^ obtained a significant decrease in the abundance of both *Cx. tarsalis* and *Cx. pipiens*, as shown by pre- and post-trapping realized inside and outside the spray zone.	[100]
New York	Controlling mosquito populations at the end of the season, before *Culex* females enter refuges, appeared to be an effective way to force declines in the virus circulation	[72]
California	This study examined the efficacy of the 2005 emergency aerial spray in Sacramento County, which used pyrethrins to control adult mosquitoes. An unsprayed area within the county was used as the control, showing a total decrease in *Cx. pipiens* and *Cx. tarsalis*, of 57.5%, compared with the pre-spray population in the treated area. They also observed a decrease in WNV infection rates in collected mosquitoes from 6.7/1,000 in the untreated areas to 3.9/1,000 in the treated areas.	[101]
Illinois	In the 2005 the city of Chicago used ground ULV treatments of sumithrin (ANVIL 10 + 10 at the dose of 1.36 g/ha), in areas with high WNV infection rates among *Culex* mosquitoes (>5 infected mosquitoes/1000). Gravid traps at 87 sites were used for monitoring. Two sequential treatments in weeks 31 and 32 decreased mean mosquito density by 54% (from 2.5 to 1.1 mosquitoes per trap-day), whereas mosquito density increased from 1.3 to 3.3 mosquitoes per trap-day at the non-sprayed sites. The difference between these changes in mosquito density was statistically significant. While other two sequential adulticide treatments in weeks 34 and 35 had no effect on mosquito density (probably because it was late in the season and the mosquitoes were presumably entering diapause and less active). Overall, there was significant decrease in mosquito density at the trap sites treated in all 4 weeks (weeks 31, 32, 34, and 35), while no significant effect was observed following single applications. Maximum likelihood estimates (MLE) of infection rate estimates varied independently of adulticide treatments, suggesting that the adulticide treatments had no direct effect on MLE. In general gravid trap counts were very low, which was probably due to large numbers of alternative oviposition sites, especially catch basins competing with the gravid traps.	[92]

Considering the efficacy of personal protection measures in reducing the risk of acquiring WNV infection Loeb [77], working in Ontario, Canada, demonstrated that the adoption of ≥ 2 personal protective behaviour attitude (such as avoidance of exposure to mosquitoes, wearing long sleeves and pants, using mosquito repellent) reduced the risk of WNV infection by half. Averett *et al*. [102] evaluated the 2003 WNV public education campaign in Kansas. Even if the campaign achieved widespread awareness among the public, people’s compliance to use protective measures was low most probably because of a low risk perception of WNV infection. The messages were most effectively delivered through television, newspapers and word-of-mouth, whereas passing messages through healthcare providers, veterinarians, magazines, and the internet were less successful. Brochures were least successful, indicating that they may be ineffective for this type of communication. None-native English speakers were less informed than native ones.

A cost-effectiveness analysis of vector control generically starts from a problem and presuppose an analysis of costs and effectiveness of each alternative solution [103]. The little scientific information focused on cost-benefit analysis in vector control targeted to WNV transmission comes from the US, following the large WNV epidemic the country experienced from 1999. The impact of WNV infection in the US was large, reaching a peak in the 2003 when 9,862 human cases (264 deaths) were registered (http://www.cdc.gov/ncidod/dvbid/westnile/surv&control_archive.htm). Zohrabian *et al*. [104] estimated the economic impact of the outbreak of WNV infection in 2002 in Louisiana, which resulted in 329 cases with 24 deaths. They considered the period June 2002-February 2003 (therefore long term costs were not considered), and included costs related to total medical care, productivity loss, public health department state activities, and vector control. Total epidemic costs were estimated as ≈ 20.14 million$ (including 9.2 million$ for mosquito control and public health agency costs) equal to 61,215 $/case. In 2005, an outbreak of WNV infection occurred in Sacramento County, California, where 163 human cases were reported. In response to WNV surveillance indicating increased WNV activity, the Sacramento-Yolo Mosquito and Vector Control District conducted an emergency aerial spray by pyrethrins for six nights on an area of ≈ 477 km^2^. The economic impact of the outbreak (including medical cost to treat cases and patients’ productivity loss) has been estimated as ≈ $2.28 million (equal to 13,987 $/case). Vector control cost was estimated as ≈ $701,790. A cost-benefit analysis indicated that only 15 cases of WNV neuroinvasive disease would need to be prevented to make the emergency spray cost-effective [105].

### Vector control decision making process

Prevention and control of WNV infection should be based on an integrated preparedness plan taking into consideration surveillance, communication, IVM activities, intersectoral collaboration, and evaluation. The activation of the plan should be based on the surveillance data from the four possible basic indicators: mosquitoes, birds, horses and humans. The main parameters to be considered are: the level of virus activity in the area estimated by the infection rate in *Culex* mosquitoes, the percentage viremic birds, the equine cases, or recent sero-conversions in sentinel birds or equines; the seasonal period when WNV activity is first detected since the early-season detection of WNV activity appears to be correlated with increased risk of human cases later in the season, whereas late-season detection of WNV activity may not indicate a real human risk. In this framework it is considered that the risk of tangential transmission exists when the surveillance activities show an increase in the circulation of the virus. The complex epidemiology of WNV infections requires that the surveillance activities are well coordinated and progressively improved based on field evidence and proper evaluation. A baseline scheme to assist an evidence based decision making process is depicted below. Difficulties in early estimation of the outbreak size in time and space complicates the implementation of adapted vector control measures, hence the need to elaborate models to achieve a more precise epidemiological forecast, allowing more effective and cost-benefit appropriate decisions (Table 3).

**Table 3:** Vector control decision making process: recommended response for the different risk levels

**Risk area**^**1**^	**Risk level**	**Probability of human outbreak**	**Description**	**Recommended response**
Predisposed	1	Unknown	Ecological condition suitable to WNV circulation AND past evidences of WNV circulation	Consider drafting WNV preparedness plan
Imperilled	2	Unknown	Ecological condition suitable to WNV circulation	Develop WNV preparedness plan, including surveillance activities and an integrated vector control plan
			AND past evidences of WNV circulation	Allocate resources necessary to enable emergency response
				Implement larval control as part of the integrated vector control in case of WNV circulation in previous year
Imperilled	3a	Low	Current surveillance findings (i.e. mosquito or birds screening) indicating WNV epizootic activity in the area, in the second part of the season (August-September-October)	As in risk level 2
				AND Implement public education programs focused on risk potential, personal protection, and emphasizing residential source reduction
				Vector control focuses on larval control
Imperilled	3b	Low to moderate	Current surveillance findings (i.e. mosquito or birds screening) indicating WNV epizootic activity in the area, in the first part of the season (May-June-July)	As in risk level 3a
				AND increase entomological and bird surveillance
				AND increase effort for public information on personal protection and continued source reduction
				AND If surveillance indicates virus circulation is increasing initiate ground adult control in areas at high risk for humans or in hot spot sites (if known)
Imperilled	4	High	WNV specific IgM detected in local non vaccinated horse(s) or WNV isolated from local horse.	As in risk level 3b
				If surveillance indicates virus circulation is increasing initiate ground adult control in areas at high risk for humans or in hot spot sites (if known)
Affected	5	ongoing outbreak, uncertainty about size	at least one human case detected (i.e. probable or confirmed human case according to EU case definition)	Response as in level 4
				AND intensify ground adult mosquito control with multiple applications in areas of high risk of human cases
				AND enhance risk communication
				AND monitor efficacy of spraying on target mosquito populations
				AND in case a large area is involved coordinate the program by an emergency unit with all authorities involved

^1^Nomenclature according to [106].

## Conclusions

Most of the available data on WNV vector control methods refer to North America, where, following the introduction of the virus in 1999 and its rapid spread through the country, an impressive amount of scientific investigation as well as organized plans and implementation of vector control activities have been developed. The reason why such an effort has never been made in Europe has obviously come from the smaller impact the WNV infection had on public health, with sporadic incursions in sensitive areas and long periods of silence and non-evident circulation. This situation seems to have changed recently with several EU countries affected in consecutive years. The US experience showed that, when applied with appropriate methodologies and resources, vector control measures such as adult control may reduce the outbreak size. Multiple sequential treatments may be necessary to reduce *Culex* female density to a level where reduction of WNV infection may be observed. If a large area is involved it becomes quite difficult to organize an effective adult control program using ground application only. The real effectiveness of mosquito larval control in preventing or reducing the risk for WNV human cases is a logical assumption but remains to be demonstrated. While preferable to adult control from the environmental points of view, larval control might not be as effective as expected due to density dependent phenomena and because it has no influence on longevity of infective mosquito females. Based on the available evidence an overview of the use of the currently existing vector control methods is presented in Table 1. Further studies are needed in the EU to assess the value of each method to prevent and control outbreaks of WNV infection and to assess the best use in time and space (e.g. by the identification of hot spot areas particularly favourable for the WNV amplification early in the season). The proposed vector control decision making process, in line with ECDC’s West Nile risk assessment tool [107], aims at supporting the rational choice of the control methods but warrants further validation and refinement based on experiences from outbreak control.

The extrapolation of information produced in North America to Europe might be limited because of the seemingly lower level of WNV transmission in the European region probably due to the long-time exposure of wild bird populations resulting in an ecosystem that is less suitable for large outbreaks. Additionally, the diversity of the susceptible bird fauna and of the vector species involved in the enzootic and tangential transmission in Europe makes WNV transmission remarkably different with the US. Aerial insecticide applications being largely adopted in the US are basically not allowed in the EU. Hence, the cost-benefit of vector control in the US compared to the EU might be very different and requires a careful specific evaluation. Therefore, there is an urgent need to analyse the European experiences on the prevention and control of outbreaks of WNV infection in order to optimize resources while keeping the risk of acquiring the infection at an acceptable level.

To be effective, vector control programs require a strong organisational backbone and co-ordination between the different stakeholders from the central to the community level relying on a previously defined plan, skilled technicians and operators, appropriate equipment, and sufficient financial resources. The permanent allocations of resources on an issue which is largely unpredictable in space and time, with elapsing periods between outbreaks that may last for years, could be difficult to sustain and justify but is needed to develop proper preparedness and response activities.

## Abbreviations

EU: European Union; B.t.i.: *Bacillus thuringiensis israelensis*; B.s.: *Bacillus sphaericus*; IVM: Integrated vector management; PBO: Piperonyl butoxide; ULV: Ultra low volume; WNV: West Nile virus.

## Competing interests

The authors declare that they have no competing interests.

## Authors’ contributions

RB carried out the literature search and prepared the first draft of the manuscript under the contract ECD.3191 financed by the ECDC. WVB contributed to writing the paper and HZ and WVB critically revised the manuscript. All authors read and approved the final submitted version.

## Authors’ information

RB is a medical entomologist currently working at the Centro Agricoltura Ambiente “G.Nicoli” in Italy, where he is the director of the Medical and Veterinary Entomology Department.

HZ is a virologist working at the European Centre for Disease Prevention and Control where he is head of the Emerging and Vector-borne Diseases programme.

WVB is a medical entomologist working at the European Centre for Disease Prevention and Control.
